# Selective Immunosuppression Targeting the NLRP3 Inflammasome Mitigates the Foreign Body Response to Implanted Biomaterials While Preserving Angiogenesis

**DOI:** 10.1002/adhm.202301571

**Published:** 2023-11-01

**Authors:** Alex H.P. Chan, Matthew J. Moore, Angus J. Grant, Yuen Ting Monica Lam, Matthew V. Darnell, Praveesuda L. Michael, Steven G. Wise, Richard P. Tan

**Affiliations:** ^1^ School of Medical Sciences Faculty of Health and Medicine University of Sydney Sydney NSW 2006 Australia; ^2^ Charles Perkins Centre University of Sydney Sydney NSW 2006 Australia

**Keywords:** biomaterials, dexamethasone, foreign body responses, implantable devices, NLRP3 inflammasome

## Abstract

Medical devices are a mainstay of the healthcare industry, providing clinicians with innovative tools to diagnose, monitor, and treat a range of medical conditions. For implantable devices, it is widely regarded that chronic inflammation during the foreign body response (FBR) is detrimental to device performance, but also required for tissue regeneration and host integration. Current strategies to mitigate the FBR rely on broad acting anti‐inflammatory drugs, most commonly, dexamethasone (DEX), which can inhibit angiogenesis and compromise long‐term device function. This study challenges prevailing assumptions by suggesting that FBR inflammation is multifaceted, and selectively targeting its individual pathways can stop implant fibrosis while preserving beneficial repair pathways linked to improved device performance. MCC950, an anti‐inflammatory drug that selectively inhibits the NLRP3 inflammasome, targets pathological inflammation without compromising global immune function. The effects of MCC950 and DEX on the FBR are compared using implanted polycaprolactone (PCL) scaffolds. The results demonstrate that both DEX and MCC950 halt immune cell recruitment and cytokine release, leading to reduced FBR. However, MCC950 achieves this while supporting capillary growth and enhancing tissue angiogenesis. These findings support selective immunosuppression approaches as a potential future direction for treating the FBR and enhancing the longevity and safety of implantable devices.

## Graphical abstract

Inhibition of the NLRP3 inflammasome using MCC950 results in similar reductions in fibrotic response compared to broad spectrum anti‐inflammatory agents such as dexamethasone. Selective targeting of NLRP3 preserves the critical elements of inflammation resolution and promotes angiogenesis and host tissue integration which is required for the long‐term functions of implanted medical device.

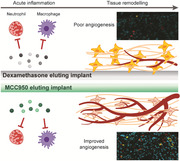

## 1 Introduction

In most clinical settings, implanted medical devices provide an immediate therapeutic impact, supplementing damaged or lost physiological functions in patients with chronic diseases. Across all implantable devices, the foreign body response (FBR) is a significant clinical concern tied to their use and requires continual observation of its impact on device efficacy and safety over time. The failure rate of all implantable devices due to the FBR is conservatively estimated to be 10%, and solving this clinical need is valued at ≈$10 billion per year.^[^ ^1^ ^]^ Typically implanted near the organ they are intended to interact with, devices are impacted by the FBR through varying degrees of implant fibrosis which can limit the proximity of the device to its target tissue. Fibrotic capsules have been shown to restrict the diffusion of analytes into the sensing elements of implanted biosensors, reducing their readout accuracies, while also impeding transduction efficiencies of electrical stimulation in microelectrode arrays implanted adjacent to nervous tissue.^[^ ^1^, ^2^ ^]^ Depending upon the device and its intended therapeutic function, FBR‐mediated fibrotic encapsulation can lead to reduced function, device failure, and/or compromised patient safety.^[^ ^3^ ^]^

Any material that is implanted into the body triggers the host inflammatory FBR. The FBR is a manifestation of the innate immune system attempting to protect the host from foreign pathogens, forming a dense collagen‐rich capsule surrounding and isolating the implant. Depending on material surface features of the device, including architecture, size, charge, and chemistry, the extent of this response can occur along a spectrum from thin and diffuse to dense and thick capsules.^[^ ^4^ ^]^ This has inspired several design and pharmacological approaches which aim to limit the severity of the FBR by antagonizing the innate immune responses that drive its progression. However, this requires a delicate balance of immunosuppression as in most instances, the success of the device also relies on tissue regeneration and local integration where the device is implanted. For example, encapsulation devices containing insulin‐secreting beta‐cells achieve optimal performance when they are minimally impacted by fibrosis, which allows the greatest amounts of insulin to leave the device.^[^ ^5^ ^]^ However, achieving therapeutic success critically relies on the regrowth of local vascular beds to ensure that the insulin can reach the systemic circulation. Adequate tissue repair involving processes such as angiogenesis and cellular regrowth are heavily reliant on innate inflammation and often suppressed by the administration of broad‐spectrum anti‐inflammatory drugs.

Current approaches largely focus on wholly suppressing innate inflammation through immunosuppressive drugs such as dexamethasone (DEX). DEX is a synthetic glucocorticoid widely used in clinical settings due to its well‐established immunosuppressive effects^[^ ^6^ ^]^ and ability to significantly reduce fibrotic capsule formation in a range of implantable devices, including glucose sensors,^[^ ^7^ ^]^ neural prosthetics,^[^ ^8^ ^]^ cochlear implants,^[^ ^8^ ^]^ vascular grafts,^[^ ^9^ ^]^ and corneal implants.^[^ ^10^ ^]^ Despite these benefits, several studies have identified important limitations of DEX. DEX also has non‐specific immunosuppressive properties, which can affect a broad range of immune cells and their related functions. In particular, DEX has been shown to inhibit the pro‐angiogenic activity of endothelial cells by downregulating VEGF and also by blocking the recruitment of macrophages which facilitate angiogenesis through cytokine release.^[^ ^11^ ^]^

An ideal drug strategy would selectively suppress the immune responses which cause the FBR while preserving those that elicit tissue healing to maintain long‐term suppression of the FBR. Mounting evidence suggests that the NLRP3 inflammasome, a key element of the innate immune system responsible for the production of cytokines implicated during the FBR may be a promising target for more specific regulation of innate inflammation. MCC950 is a selective small molecule inhibitor of the NLRP3 inflammasome that reduces the production of pro‐inflammatory cytokines without impacting other innate inflammasomes.^[^ ^12^ ^]^ In wound healing studies, MCC950 has demonstrated no impairments to native angiogenesis,^[^ ^13^ ^]^ suggesting that the selective functions of MCC950 can carry robust anti‐inflammatory actions without the anti‐angiogenic limitations of DEX. Only a single recent study to date using neuronal implants has shown that the use of MCC950 hinders the foreign body reaction while leaving nerve regeneration unaffected.^[^ ^14^ ^]^ These promising findings inspire further exploration of MCC950 during the FBR and the concept of selective immunosuppression, which may help potentially identify distinct tissue repair processes more generally within the body not specific to the central nervous system.

**Figure 1:**
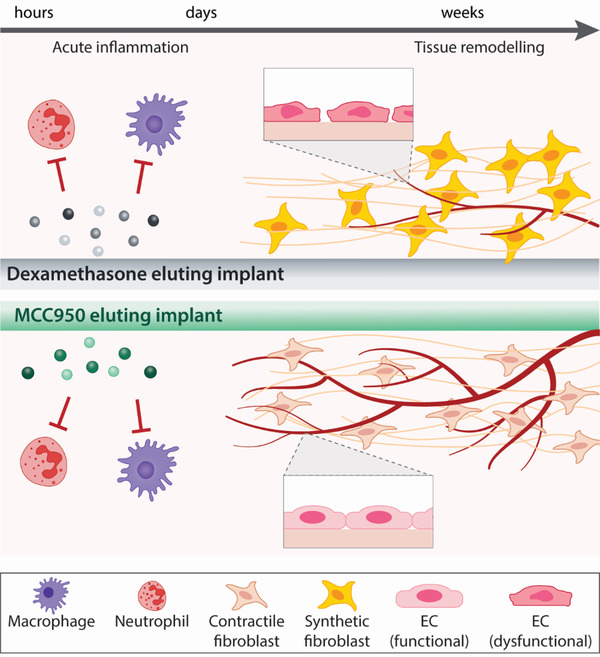
Schematic comparison of dexamethasone (DEX) and MCC950 as pharmacological approaches to mitigating the foreign body response (FBR). DEX and MCC950 exhibit similar immunosuppressive effects in the acute stages of the FBR, reducing the recruitment of neutrophils and macrophages. End‐stage remodeling outcomes show both drugs can effectively reduce fibrotic capsule formation, however MCC950 shows evidence of increased suppression of collagen‐producing (synthetic) fibroblast differentiation. Importantly these effects of MCC950 occur without compromising endothelial integrity/function and preserves angiogenesis. Abbreviations: EC, endothelial cell.

In this study, we conduct a comparative analysis of MCC950 against DEX as an alternative immunosuppressive approach for mitigating the FBR to subcutaneous implants. We first show in vitro, that although DEX is a more potent anti‐inflammatory drug, MCC950 does not impair endothelial cell integrity and function. Further evaluation in vivo using a 14 day mouse subcutaneous implantation model of PCL scaffolds showed that both DEX and MCC950 equally suppressed fibrotic capsule formation, however MCC950 significantly enhanced stable angiogenesis around the implanted scaffolds. Further examination of these effects in a 14 day transgenic mouse model which allows non‐invasive tracking of immune cell populations from the bone marrow, demonstrated that while MCC950 enhanced the recruitment of immune cells, it showed the greatest reduction in capsule development and enhanced vascular repair, suggesting the enhancement of “reparative” inflammation (**Figure** **1**). These findings support NLRP3 inhibition as an alternative and selective anti‐inflammatory drug approach for the improved performance and safety of implanted medical devices and materials.

## 2 Results

### 2.1 Macrophage Activation

**Figure 2:**
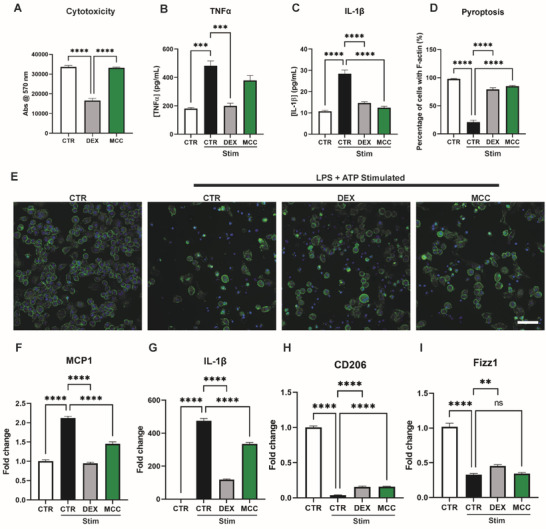
Immunosuppresive effects of DEX and MCC950 on cultured J774a.1 murine macrophages. A) alamarBlue cytotoxicity assay following 3 day culture with DEX and MCC950. B,C) Levels of TNF‐α and IL‐1β cytokines following NLRP3 inflammasome stimulation (STIM; LPS+ATP) measured by ELISA. D,E) Quantification and representative images of pyroptosis. Cells stained with DAPI (blue) and rhodamine phalloidin (green) to visualise cell nuclei and F‐actin, respectively. Data represented as percentage of cells with concentric F‐actin membrane. F–I) qPCR of M1 (MCP1, IL‐1β) and M2 (CD206, Fizz1) gene sets 24 h post‐stimulation (STIM; LPS+ATP). Data represented as mean ± SEM (*n* = 3–4). Statistical significance was determined using Dunnett's multiple comparison one‐way ANOVA test relative to stimulated group (**p < 0.01, ***p < 0.001, ****p < 0.0001). Scale bar represents 50 µm.

The anti‐inflammatory effects of DEX and MCC950 were evaluated in murine macrophages. AlamarBlue cytotoxicity assays after 3 days showed that DEX significantly decreased macrophage viability (33 598 ± 759 vs 16 572 ± 1089, *p* < 0.0001), whereas MCC950 has no impact on viability compared to untreated controls (33 598 ± 759 vs 33 178 ± 299, *p* = 0.9023) (**Figure** **2**A).

An in vitro model of NLRP3 inflammasome‐mediated immune activation was used to investigate selectivity of DEX and MCC950 anti‐inflammatory effects. ELISA was used to quantify the secretion of TNF‐α and IL‐1β as non‐specific and specific downstream products of the NLRP3 inflammasome, respectively (Figure 2B,C). DEX significantly reduced the secretion of both TNF‐α and IL‐1β compared to stimulated controls (199.8 ± 18.73 pg mL^−1^, 59% reduction and 14.67 ± 1.18 pg mL^−1^, 52% reduction respectively). However, MCC950 only reduced the secretion of IL‐1β, but not TNF‐α (12.47 ± 1.18 pg mL^−1^, 48% reduction). This suggested that DEX had broader anti‐inflammatory functions compared to MCC950 which was selective only to NLRP3 inflammasome specific cytokine production.

Macrophage cultures were then stained for actin to measure the degree of pyroptosis following drug treatment. Both DEX and MCC950 decreased pyroptosis whereby the percentage of cells with intact cytoskeleton were significantly greater than stimulated controls (79.18 ± 2.9% and 84.81 ± 1.4% compared to 20.82 ± 2.6%) (Figure 2D,E).

To examine any direct effects of either drug on macrophage polarization, qPCR was performed on M1 and M2‐related gene expression. These genes were chosen based on their established reliability as M1 and M2 markers based on past studies examining M1/M2 polarization in vitro. MCP1 and IL‐1β are classical M1 markers.^[^ ^15^ ^]^ CD206 and Fizz1 are classical M2 markers.^[^ ^16^ ^]^ Within the M1 gene set, both DEX and MCC950 significantly reduced MCP‐1 and IL‐1β gene expression compared to stimulated control groups (Figure 2F,G). However, this reduction was greater with DEX treatment. DEX caused a 55% and 75% reduction compared to a 32% and 30% reduction with MCC950 in MCP‐1 and IL‐1β, respectively. Within the M2 gene set, DEX significantly increased CD206 and Fizz1 gene expression relative to stimulated controls (Figure 2H,I). However, in MCC950 groups, only increases in CD206 were observed. This validated that DEX possessed more robust and broad‐acting immunosuppressive behavior, compared to the selective functions of MCC950. Additionally, while both drugs facilitated upregulation of some M2 gene transcription relative to stimulated controls, this failed to reach untreated control levels, suggesting that both drugs acted primarily to suppress pro‐inflammatory M1 activity in macrophages rather than enhancing anti‐inflammatory M2 functions.

### 2.2 Myofibroblast Differentiation

Fibroblast to myofibroblast differentiation, indicated by an increase in smooth muscle cell alpha actin (SMα actin) and Collagen I (Col I) expression, precedes collagen deposition within the fibrotic capsule. The anti‐fibrotic effects of DEX and MCC950 were evaluated using an in vitro model of myofibroblast differentiation by TGF‐β stimulation.^[^ ^17^ ^]^

**Figure 3:**
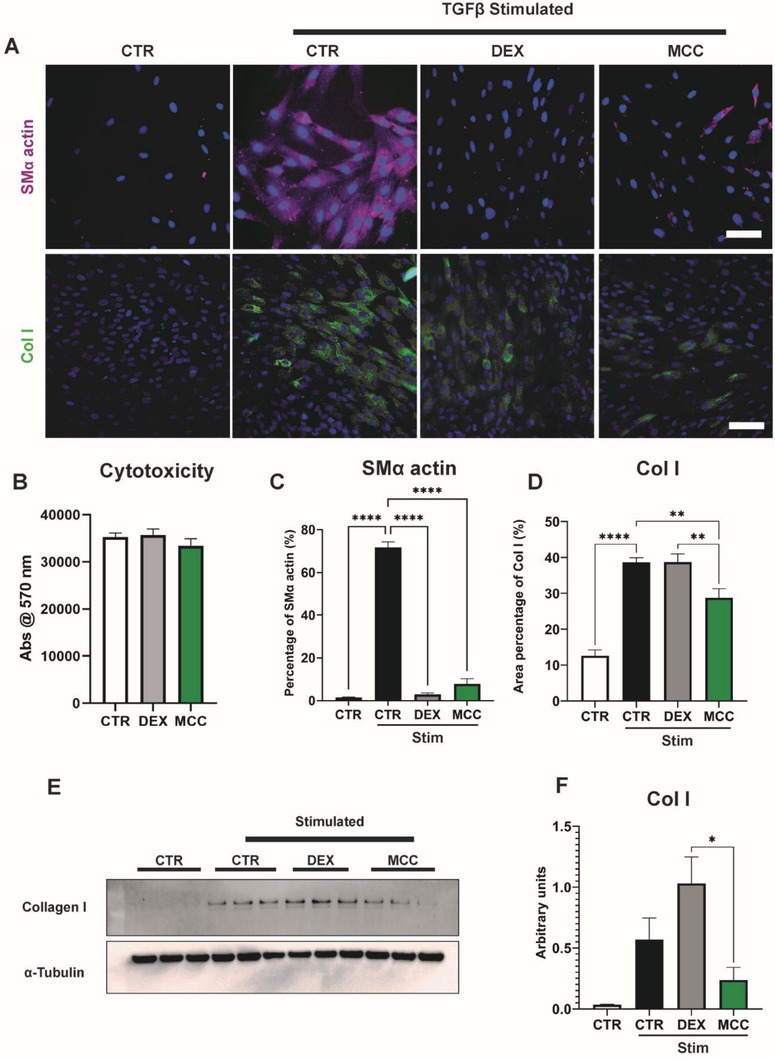
MCC950 possessed anti‐fibrotic effects on human dermal fibroblasts. A) Representative images of human dermal fibroblasts stimulated with TGF‐β to induce fibroblast‐to‐myofibroblast differentiation, indicated by increased SMα actin (purple) and Collagen I (Col I, green) intracellular staining. Cells counterstained using DAPI (blue). B) alamarBlue cytotoxicity assay following 3 day culture with DEX and MCC950. C,D) Quantification of intracellular staining of SMα actin and Col I in fibroblasts after 3 days of TGF‐β stimulation in the presence of DEX and MCC950. E,F) Western Blots and quantification of Col I. Data represented as mean ± SEM (*n* = 3–4). Statistical significance was determined using Dunnett's multiple comparison one‐way ANOVA test relative to stimulated group (*p < 0.05, **p < 0.01, ****p < 0.0001). Scale bar represents 100 µm.

Immunocytochemistry staining for SMα actin and Col I showed that expression of both proteins was increased throughout the cell cytoplasm following TGF‐β stimulation. Both DEX and MCC950 showed reduced SMα actin after stimulation, returning to levels similar to untreated controls levels (**Figure** **3**A). For Col I staining, DEX had no effect following stimulation, whereas MCC950 reduced levels back to control (Figure 3A). Complementary alamarBlue assays showed that these effects were independent of any potential cytotoxicity effects as neither drug decreased cell viability (Figure 3B). Quantification of SMα actin stains showed a nearly 95% and 89% reduction by DEX and MCC950 relative to stimulated controls, respectively (Figure 3C). Quantification of Col I showed no significant difference between DEX and stimulated controls, as opposed to MCC950 which showed a 26% reduction (28.74 ± 2.6% vs 38.71 ± 1.2%) (Figure 3D).

As smaller reductions were observed in Col I under both drug treatments relative to SMα actin, Western Blots were performed to further quantify levels of Col I (Figure 3E,F). MCC950 reduced Col I expression by 58% compared to stimulated control (0.24 ± 0.10 vs 0.57 ± 0.18), whereas DEX showed an 80% increase in relative expression of Col I (1.03 ± 0.22 vs 0.57 ± 0.18) (Figure 3F). Together, this suggests that MCC950 was not only preventing myofibroblast differentiation but had more robust inhibitory effects on downstream collagen production.

### 2.3 Endothelial Cell Integrity and Function

**Figure 4:**
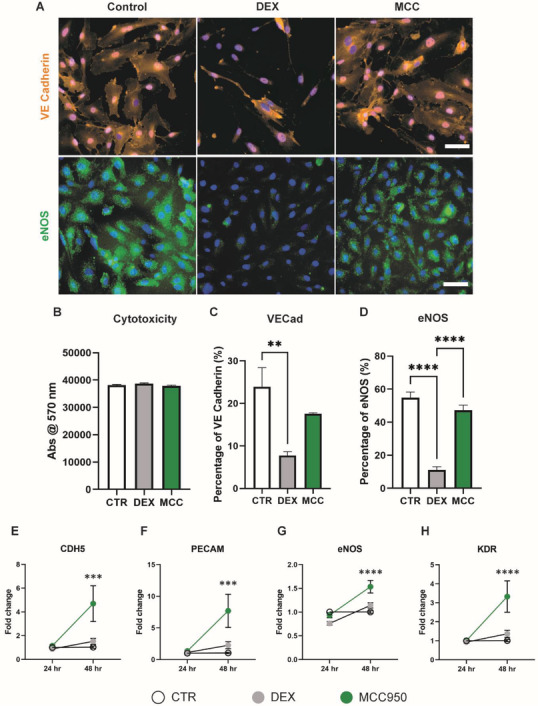
MCC950 promoted integrity and function of human endothelial cells. A) Representative images of endothelial cells stained for vascular endothelial‐cadherin (VE‐cadherin, orange) and endothelial nitric oxide synthase (eNOS, green) after 3 days in culture with DEX and MCC950. Cells counterstained using DAPI (blue). B) alamarBlue cytotoxicity assay following 3 day culture with DEX and MCC950. C,D) Quantification of VE‐cadherin and eNOS staining following 3 day treatment with DEX and MCC950. E–H) qPCR of pro‐angiogenic gene set (CDH5, PECAM, eNOS, and KDR) 24 and 48 h post‐stimulation with DEX and MCC950. Data represented as mean ± SEM (*n* = 3–4). Statistical significance was determined using Dunnett's multiple comparison one‐way ANOVA test relative to stimulated group (**p < 0.01, ***p < 0.001, ****p < 0.0001). Scale bar represents 50 µm.

The direct effects of DEX and MCC950 on endothelial cells were examined to distinguish potential differences in angiogenesis. Endothelial cells were treated with both drugs for 3 days and stained for the endothelial integrity and function markers, vascular endothelial cadherin (VE‐cadherin) and endothelial nitric oxide synthase (eNOS), respectively. Immunocytochemistry stains for both markers showed decreased levels and spreading within the cytoplasm when treated with DEX (**Figure** **4**A). Complementary alamarBlue assays showed that this was not due to cytotoxicity effects as neither drug impacted endothelial cell viability (Figure 4B). Quantification of both VE‐Cad and eNOS markers showed that DEX caused a 68% and 80% reduction, respectively, compared to untreated controls, whereas MCC950 showed no significant changes in expression of either marker (Figure 4C,D).

To further study the transcriptional changes responsible for these effects, qPCR was conducted for a classical array of pro‐angiogenic endothelial genes including VE‐cadherin (CDH5), PECAM, eNOS, and VEGF receptor 2 (KDR) across the first 48 h in culture (Figure 4E–H). DEX showed no significant upregulation of any of these genes relative to untreated control across the 48 h period. In contrast, MCC950 caused significant fold changes by 48 h compared to control for all genes within the set; CDH5: 4.70 versus 1.03, PECAM: 7.69 versus 1.04, eNOS: 1.53 versus 1.00, and KDR: 3.33 versus 1.01. This suggested that MCC950 was actively supporting angiogenic activity at the transcriptional level.

### 2.4 Cellular Recruitment In Vivo

**Figure 5:**
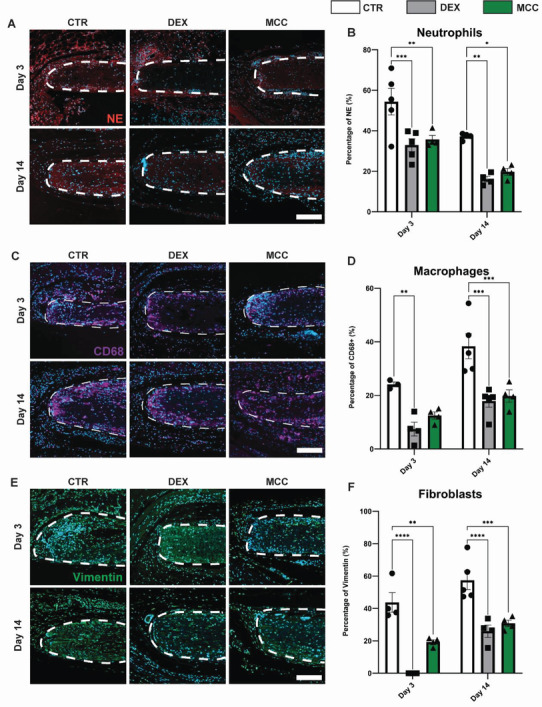
MCC950 and DEX possessed similar levels of immunosuppressive and anti‐fibrotic effects on subcutaneously implanted PCL scaffolds at 3 and 14 days post‐implantation. A) Representative images of neutrophils stained with neutrophil elastase (red), counterstained for nuclei with DAPI (blue). B) Quantification of area stained with neutrophil elastase. C) Representative images of macrophages stained with CD68 (purple), counterstained for nuclei with DAPI (blue). D) Quantification of area stained with CD68. E) Representative images of fibroblasts stained with vimentin (green), counterstained for nuclei with DAPI (blue). F) Quantification of area stained with vimentin. Data represented as mean ± SEM (*n* = 4–5). Statistical significance was determined using Tukey's multiple comparison two‐way ANOVA test relative to control group (*p < 0.05, **p < 0.01, ***p < 0.001, ****p < 0.0001). Scale bar represents 100 µm.

DEX and MCC950 were next evaluated in vivo as treatments on subcutaneously implanted PCL scaffolds for 14 days. Acute and sub‐acute immune responses were assessed by staining for neutrophils and macrophages, respectively. Neutrophils were found to localize largely around the periphery of the scaffolds (**Figure** **5**A). Quantification showed that neutrophil recruitment generally resolved over the 14‐day implantation period in controls. DEX and MCC950 enhanced this resolution to similar levels with a roughly 39% and 34% reduction (32.9 ± 3.2% and 35.8 ± 1.9% vs 54.4 ± 6.55%) at day 3, 57% and 47% reduction (16.1 ± 1.4% and 19.7 ± 1.7% vs 37.2 ± 0.8%) compared to control at days 14 (Figure 5B).

In contrast to neutrophils, macrophages were found both at the scaffold periphery and within the implant (Figure 5C). Quantification showed that macrophage recruitment generally increased in control scaffolds over 14 days. DEX treatment reduced macrophage recruitment at both days 3 and 14 compared to controls (7.43 ± 2.6% vs 24.1 ± 0.9% and 17.9 ± 2.2% vs 38.3 ± 4.67%) (Figure 5D). However, MCC950 only showed significant decreases at day 14 compared to control (19.8 ± 2.3% vs 38.3 ± 4.67%) which were of a comparable magnitude to DEX.

Fibroblast recruitment was also assessed to determine cellular changes to scaffold remodeling. Similar to macrophages, fibroblasts were found both within the scaffold periphery and inside (Figure 5E). Quantification showed that recruitment increased over the 14 days with DEX treatment reducing recruitment at days 3 and 14 (0.0 ± 0.0% vs 43.7 ± 6.0% and 25.9 ± 3.8% vs 57.4 ± 5.8%). MCC950 only showed significant reductions at day 14 compared to control (30.8 ± 2.0% vs 57.4 ± 5.8%), which were again comparable to DEX.

### 2.5 Scaffold Remodeling In Vivo

**Figure 6:**
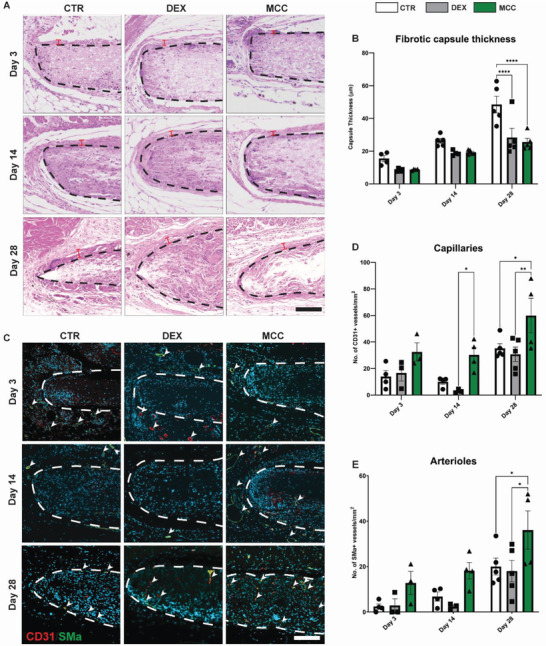
MCC950 and DEX equally suppressed fibrotic capsule formation, however only MCC950 enhances angiogenesis around subcutaneously implanted PCL scaffolds at 3, 14, and 28 days post‐implantation. A) Representative images of implant cross‐sections (black dotted line) stained H&E to visualize fibrotic capsule (red bars). B) Quantification of fibrotic capsule thickness. C) Representative images of scaffold cross‐sections co‐stained with CD31 (red) and SMα actin (green). Cell nuclei counterstained with DAPI (blue). D) Quantification of capillaries mm^−2^ indicated by CD31 staining alone. E) Quantification of arterioles mm^−2^ indicated by co‐staining of CD31 and SMα actin. Data represented as mean ± SEM (*n* = 4–5). Statistical significance was determined using Tukey's multiple comparison two‐way ANOVA test relative to control group (*p < 0.05, **p < 0.01, ***p < 0.001). Scale bar represents 100 µm.

End stage scaffold remodeling outcomes were assessed by measuring fibrotic capsule thickness and levels of local angiogenesis. H&E staining of the implanted scaffolds showed increasing capsule thickness and cell infiltration into the scaffold body over 28 days (**Figure** **6**A). Quantification showed increasing capsule thickness over 28 days in controls, while both DEX and MCC950 showed the similar levels of reduction in capsule thickness at days 3 (8.4 ± 0.5 µm and 8.7 ± 0.2 µm vs 15.4 ± 1.9 µm) and 14 (18.8 ± 0.8 µm and 19.3 ± 0.6 µm vs 26.3 ± 1.5 µm), then significant reductions at 28 (18.8 ± 0.8 µm and 19.3 ± 0.6 µm vs 26.3 ± 1.5) (Figure 6B). Collagen content was further assessed within the fibrotic capsule using Masson's staining which showed increasing collagen content over time, but no significant differences were observed between DEX and MCC groups compared to control (Figure S2, Supporting Information). To assess whether the proximity of the scaffolds influenced local drug release due to potential cross‐reactivity with adjacent scaffolds, we conducted a separate cohort of mice at day 14. In this experiment, all scaffold groups were rotated to allow implantation in all three scaffold locations. Similar trends were observed in capsule reduction, suggesting that the effects of drug treatments remained localized to the implant site (Figure S3, Supporting Information).

The levels of local angiogenesis were determined by quantifying the number of blood vessels co‐stained with CD31^+^ and SMα actin. Most vessels were observed in the periphery of the scaffolds (Figure 6C). The endothelial marker CD31^+^ alone was used as a measure of capillary formation whereas co‐staining with SMα actin indicated the formation of more stable, mature arterioles. Capillary formation decreased in controls over 14 days in both control and DEX groups (Figure 6D). However, in MCC950 groups, high levels of capillary formation were sustained from day 3 to 28. We observed significant differences at day 14, where MCC950 had an 874% increase in capillary formation compared to DEX, respectively (30.2 ± 5.6 counts mm^−2^ vs 3.1 ± 1.3 counts mm^−2^). At day 28, MCC950 had a 70% and 95% increase in capillary formation compared to control and DEX (59.9 ± 13.0 counts mm^−2^ vs 35.1 ± 3.6 counts mm^−2^ and 30.7 ± 5.5 counts mm^−2^). Similar trends were observed in arteriole formation where MCC950 sustained high levels from day 3 to 14 (Figure 6E). By day 28, MCC950 had significantly increases in arterioles, 80% and 100% increase compared to control and DEX, respectively (36.1 ± 8.4 counts mm^−2^ vs 20.0 ± 3.7 counts mm^−2^ and 18.0 ± 4.7 counts mm^−2^). Together these findings supporting that the selective immunosuppression of MCC950 possessed similar anti‐fibrotic effects as DEX but with the added advantage of enhanced stable angiogenesis.

### 2.6 Regenerative Immune Cell Tracking

**Figure 7:**
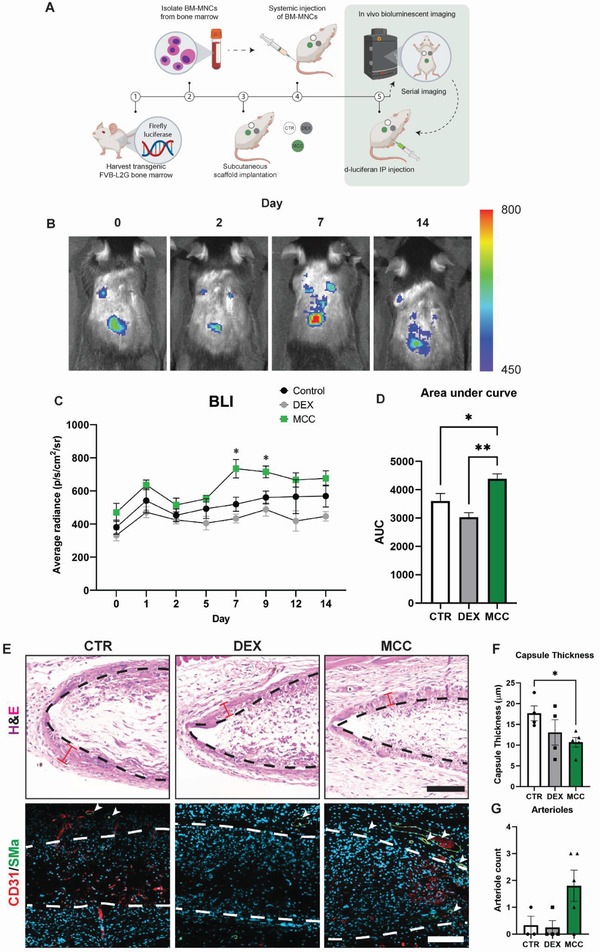
MCC950 enhanced the engraftment of injected immune cell fractions yet had the thinnest fibrotic capsules and highest levels of native arteriole formation around PCL scaffolds subcutaneously implanted for 14 days. A) Schematic representation of systemic (tail‐vein) injected bioluminescent (BLI) bone marrow mononuclear cells (BM‐MNCs). B) Representative BLI images of mouse backs with subcutaneously implanted scaffolds at days 0, 2, 7, and 14. Heat bar represents BLI signal in units of radiance (p s^−1^ cm^−2^ sr^−1^). C) Temporal measurements of BLI signal over 14 days across control, DEX and MCC950 implants. Statistical significance was determined using two‐way ANOVA test relative to control group (*p < 0.05). D) Representation of temporal measurements as area under the curve (AUC) over 14 days. E) Representative images of H&E, and CD31 (red)/SMα (green) stained scaffold cross sections at 14 days post‐implantation and BM‐MNC injection. Dotted lines represent scaffold borders. Red bars indicated fibrotic capsule thickness. Quantification of F) fibrotic capsule thickness and G) native arteriole count mm^−2^. Data represented as mean ± SEM (*n* = 4–5). Statistical significance was determined using Dunnett's multiple comparison one‐way ANOVA test relative to control group (*p < 0.05, **p < 0.01). Scale bar represents 100 µm.

To more closely examine these findings, the same scaffold groups were implanted into a 14 day model of bioluminescent cell tracking using injected transgenic bone marrow‐mononuclear cells (BM‐MNC) (**Figure** **7**A). As we have established in prior work, this model allows for non‐invasive tracking of BM‐MNC, a well‐established immune cell fraction from the bone marrow consisting largely of innate immune cells combined with small numbers of stem cell precursors (mesenchymal, endothelial, and hematopoietic).^[^ ^18^ ^]^ Mice were systemically tail vein injected with BM‐MNC after scaffold implantation and serially imaged over 14 days and bioluminescent images recorded to visualize the BM‐MNC homing and engraftment (Figure 7B). Bioluminescent signal was observed primarily at the scaffold implantation sites and was largest for MCC950 scaffolds. When plotted over time, as expected, DEX showed lowered levels of BM‐MNC recruitment compared to control (Figure 7C). In contrast, MCC950 showed enhanced levels of BM‐MNC recruitment, with the largest separation occurring at day 7. Quantification of area under the curve confirmed that MCC950 had ≈22% and 45% increase in BM‐MNC recruitment over the 14 days compared to control and DEX, respectively (4390 ± 171 vs 3608 ± 262 and 3031 ± 160) (Figure 7D).

Complementary histology was conducted to determine macrophage phenotype and remodeling outcomes of the scaffolds at 14 days. M1 and M2 phenotype analysis showed trends in MCC950 groups with reduced M1 phenotypes and elevated M2/M1 ratios, however neither of these reached statistical significance (Figure S4, Supporting Information). Further H&E staining confirmed that despite the enhanced BM‐MNC recruitment, MCC950 scaffolds still had the thinnest fibrotic capsules across all groups (Figure 7E,F). Quantification of native arteriole formation also revealed that MCC950 had regenerated more native skin arterioles in the surrounding scaffold periphery (Figure 7E,G). These findings further validated the unique selective immunosuppression functions of MCC950 to halt fibrotic capsulation formation while enhancing pro‐angiogenic tissue repair.

## 3 Discussion

The limitations of DEX as a broad spectrum anti‐inflammatory suppressor of the FBR to implanted materials and devices have highlighted the trade‐off in mitigating fibrotic capsule development. While suppressing the innate immune system largely prevents fibrotic encapsulation of devices, it often comes at the cost of tissue repair, which is reliant on inflammation and is equally critical to the successful host integration of the device and its long‐term performance.^[^ ^19^ ^]^ Moreover, the reported transient nature of DEX on delaying fibrotic responses suggests that promoting tissue repair may be a more effective long‐term strategy for preventing the FBR.^[^ ^20^ ^]^ Here, we investigated whether this balance of selective immunosuppression could be achieved through antagonism of the NLRP3 inflammasome. We conducted a detailed comparative analysis of the small molecule NLRP3 inhibitor, MCC950, relative to DEX to determine if this targeted anti‐inflammatory approach is a potentially superior immunosuppressive strategy to broad‐spectrum antagonism during the FBR.

Within macrophage cultures in vitro, DEX was a more broad‐acting immunosuppressant, decreasing macrophage proliferation and inhibiting both classical and NLRP3‐specific cytokine secretion of TNF‐α and IL‐1β, respectively. In contrast, MCC950 function appeared specific to NLRP3 inflammasome‐mediated production of IL‐1β. These findings are a validation of the well‐established selective immunosuppressive features of MCC950.^[^ ^21^ ^]^ NLRP3 inflammasome activation triggers the programmed cell death pathway of pyroptosis, which is used as a suitable indicator of macrophage activation in the context of the FBR. Pattern recognition receptors (PRRs) triggered either by damage‐associated molecular patterns (DAMPs) released by cells damaged during material implantation,^[^ ^22^ ^]^ or directly activated when macrophages first adhere to the material surface, leads to IL‐1β secretion and pyroptosis.^[^ ^23^ ^]^ MCC950 showed rescue of pyroptosis comparable to DEX, indicating that selective immunosuppression was just as robust as a broad‐spectrum approach. However, the mechanisms underlying these effects within macrophages, specifically toward phenotype polarization, are still not completely understood. Contrasting studies examining MCC950 in myocardial infarction showed increased M2 polarization,^[^ ^24^ ^]^ while those in wound healing showed MCC950 had no effect on M2 polarization,^[^ ^13^ ^]^ suggesting that MCC950 effects on M2 polarization may be context dependent. In support of the latter, our examination of MCC950 at the transcriptional level show that the direct functions of MCC950 on macrophages are more centered around antagonizing M1 pro‐inflammatory gene expression rather than upregulating M2 anti‐inflammatory genes. This suggests that in the FBR setting, MCC950 does not actively enhance tissue healing but instead provides immune conditions which allow native repair processes to occur. Our data show this is by antagonizing M1 pathways similar to DEX, or possibly through direct effects on other cells involved in tissue repair.

This concept was further explored by investigating the direct effects of DEX and MCC950 on dermal fibroblasts using a model of TGF‐β mediated myofibroblast differentiation.^[^ ^17^ ^]^ Both DEX and MCC950 appeared to inhibit myofibroblast differentiation, indicated by reduced SMα actin expression. Importantly this was not due to adverse effects on cell viability. However, the largest discrepancy observed between DEX and MCC950 was found in the expression of collagen type I (Col I). While SMα actin is an indicator of the contractile phenotype, Col I is the major product of this phenotype, providing structural support and serving as the major constituent of the fibrotic capsule.^[^ ^25^ ^]^ As such, Col I serves as a critical measure of anti‐fibrotic function, as a reduction in phenotype does not necessarily directly correlate to functional changes. To our knowledge, these effects of MCC950 are the first to demonstrate a reduction of Col I in myofibroblasts and hold promising implications for inhibiting fibrotic capsule development. This would present in vivo as more robust anti‐fibrotic effects compared to DEX and/or impact the composition of fibrotic capsules through thinner capsules which may permit increased angiogenesis.

This aligned positively with our endothelial assays demonstrating that MCC950 had no damaging consequences on endothelial cells. Consistent with literature, DEX directly impeded the integrity and function of endothelial cells,^[^ ^26^ ^]^ marked by reductions in VE‐cadherin and eNOS protein expression, respectively. In contrast, MCC950 showed no impact on these protein expression levels. Instead, surprisingly at the transcriptional level MCC950 appeared to have enhanced pro‐angiogenic effects upregulating CDH5, PECAM, KDR, and eNOS gene expression. These effects of MCC950 are in contrast to recent findings in wound healing studies that show MCC950 does not impact angiogenesis^[^ ^13^ ^]^ or in ischemic retinopathy models that show MCC950 inhibits neovascularization.^[^ ^27^ ^]^ While the role of NLRP3 in endothelial cells is still not clearly understood, our findings suggest a direct link to both function and integrity in an implant fibrosis setting. Acknowledging the different tissue settings of the prior studies, our results provide some of the first evidence that MCC950 can enhance pro‐angiogenic signaling in an implant context.

In vivo, DEX and MCC950 appeared to have a similar magnitude of effect on reducing both immune cell and fibroblast recruitment toward implants. This was somewhat surprising, given that DEX has a well‐documented robust and broad‐spectrum anti‐inflammatory function, targeted NLRP3‐inflammasome suppression would have been expected to have a reduced effect. This concept has been recently explored by Barone et al. in the context of neuron implants where both DEX and MCC950 were observed to decrease the fibrotic capsules to similar magnitudes when compared to non‐treated control groups.^[^ ^14^ ^]^ However, in contrast to our study, both DEX and MCC950 were loaded at doses of 25 mm compared to our scaffolds that were given 2.5 mm, a dose tenfold less. Despite this disparity in dosing, our reductions in capsule thickness of ≈27% by day 28 is comparable to reductions reported by Barone et al. of ≈29%. It is important to note however differences in drug delivery that was evaluated over 3 months in that study and was achieved through a slow release PDMS scaffold which possesses both a different architecture and chemical profile to our electrospun PCL scaffolds. Nevertheless, our observation of similar responses in fibrotic capsule reduction by MCC950 is supported by increasing evidence demonstrating that NLRP3‐mediated inflammation is responsible for the large majority of innate immune responses which drive the FBR.^[^ ^28^ ^]^ More broadly, this would suggest that antagonism of NLRP3 could offer selective inhibition of the FBR, without compromising global innate immune signaling that would otherwise be involved in tissue repair.

This is strongly evidenced by the observed scaffold remodeling responses which revealed that DEX and MCC950 equally reduced capsule thickness, however MCC950 was found to also enhance both capillary and arteriole formation around implanted scaffolds. Immune cell migration to implanted materials and ensuing angiogenesis are linked processes during the FBR. The presence of a foreign material triggers an inflammatory response, recruiting immune cells to the site. These cells release pro‐inflammatory cytokines such as IL‐1 and TNFα,^[^ ^29^ ^]^ which stimulate the release of angiogenic growth factors, promoting new blood vessel formation. This is vital for supplying oxygen and nutrients to the area, aiding in the resolution of inflammation as well as tissue repair. Our findings suggested that the immunosuppressive effects of MCC950 were linked to direct pro‐angiogenic effects in agreement with our in vitro endothelial cell results. There is evidence to suggest that the NLRP3 inflammasome is expressed in endothelial cells, although the extent of its expression and its role in these cells is still not fully understood. Studies have shown that NLRP3 can be activated in endothelial cells in response to stimuli common to cardiovascular pathologies, such as high blood glucose levels and oxidized low‐density lipoprotein (oxLDL).^[^ ^30^ ^]^ Endothelial NLRP3 activation promotes canonical secretion of cytokines IL‐1β and IL‐18 which induce endothelial dysfunction.^[^ ^31^ ^]^ Extending from the positive benefits of MCC950 on enhanced angiogenesis, our findings warrant further research into the specific triggers of the NLRP3 activation in endothelial cells as well as in varying contexts such as the FBR.

The selective immunosuppressive functions of MCC950 were further exemplified in a BM‐MNC tracking model. Previous characterization of this model showed that BM‐MNC are a regenerative cell fraction largely comprised of CD11b^+^/CD45^+^ immune cells.^[^ ^18^ ^]^ As expected, DEX showed reduced engraftment of BM‐MNC, indicative of suppressed immune cell recruitment and translating to reduced capsule thickness. Surprisingly, MCC950 showed increased BM‐MNC engraftment yet had the smallest fibrotic capsules. Additionally, MCC950 scaffolds were found to have more native sized vessels commonly found in cutaneous tissue. Whether or not enhanced angiogenesis here is the cause of increased BM‐MNC engraftment or a product of it has yet to be determined. Nevertheless, these outcomes suggest that the immune cells being recruited as a result of MCC950 treatment, were phenotypes not involved in capsule development but rather angiogenesis. This highlights that distinct immune pathways were responsible for both fibrotic capsule development and tissue repair, and further MCC950 was selectively enhancing only those associated with repair. Further investigation into macrophage phenotype, however, showed no significant differences in M2 phenotypes with MCC950, an outcome that aligned with our previous macrophage in vitro findings. This implies that more detailed mechanistic studies are necessary to fully comprehend the immune pathways involved in tissue repair and/or the field is yet to identify new reparative M2‐like signaling pathways that are active during the FBR but not accurately detected by conventional M2 markers such as CD206 and MHC II. Overall, increased BM‐MNC engraftment was consistent with findings from our previous characterization paper using this model, which correlated enhanced BM‐MNC scaffold engraftment with improved in situ regeneration, demonstrating enhanced tissue repair with MCC950.

## 4 Conclusion

Our work challenges the notion that all inflammation is harmful during the FBR while highlighting the limitations of broad‐spectrum anti‐inflammatory approaches like DEX. The findings in this study provide evidence for the occurrence of differential immune responses as a result of MCC950 treatment during the FBR. The selective immunosuppressive approach of MCC950 achieves similar outcomes to DEX with regard to implant fibrosis yet preserves tissue repair processes involved in angiogenesis. These results highlight the potential of NLRP3 inhibition as an alternative therapeutic target for improved long‐term performance of implanted materials and devices.

## 5 Experimental Section

### Cell Culture

J774a.1 mouse macrophages (Sigma Aldrich, MA, USA), human dermal fibroblasts (hFB, ThermoFisher Scientific, Waltham, MA, USA), and human coronary artery endothelial cells (hCAECs, Cell Applications, San Diego, CA, USA) were cultured in a CO_2_ (5%) humidified incubator at 37 °C. Macrophages and hFB were cultured in Dulbecco's Modified Eagle Medium (DMEM; Gibco, Carlsbad, CA, USA) supplemented with fetal bovine serum (FBS) (10% v/v) and antibiotics (100 U mL^−1^ penicillin and 100 µg mL^−1^ streptomycin). hCAECs were cultured in MesoEndo medium (Merck, 212‐500, Kenilworth, NJ, USA) without additional supplements. The culture media was refreshed every 2–3 days, and cells were subcultured after reaching ≥85% confluency.

### Cytotoxicity

Dexamethasone (DEX) and MCC950 (MCC) were sourced as water soluble formulations (Sigma) to maintain consistent buffer conditions throughout the study. The cytotoxicity of DEX and MCC950 on macrophages, hFBs and hCAECs was assessed via cell viability assays. Cells were seeded into 96‐well plates (2000 cells per well) and cultured in their respective media. 24 h post seeding, cells were treated with each drug at concentrations of 0.5, 5, and 50 µm, then incubated for 3 days. Cell viability was determined using alamarBlue cell viability reagent (ThermoFisher Scientific, Waltham, MA, USA). Cells were cultured in alamarBlue reagent mixed 1:9 with culture media for 2 h. Fluorescence was measured using a Tecan M‐1000 plate reader (Ex. 560/Em. 590 nm). Cell viability was recorded as a relative measurement against the untreated control group. From this concentration curve, 50 µm was determined to be the concentration used for all subsequent assays (Figure S1, Supporting Information).

### Macrophage Stimulation

Pyroptosis was determined by assessing degradation of F‐actin staining in J774a.1 cells. Cells were seeded into 96‐well plates (2 × 10^4^ cells per well) and liposaccharide (LPS) (1 µg mL^−1^) was immediately added to the culture media. After 90 min of LPS stimulation, cells were treated with adenosine triphosphate (ATP) (1.25 mm) and either DEX or MCC (both 50 µm), then incubated for 24 h. Cells were fixed and stained for F‐actin with phalloidin.

Release of TNF‐α and IL‐1β from stimulated J774a.1 mouse macrophages were measured by ELISA. Following the same stimulation protocol, cell supernatants were collected 24 h after stimulation and analyzed using ELISA kits (Abcam, Cambridge, UK) for TNF‐α (ab208348) and IL‐1β (ab197742) per manufacturer instructions.

### Quantitative PCR

Gene expression of macrophage and endothelial cells was assessed using qPCR. For macrophage, J774a.1 mouse macrophages were seeded on 6 well plates and stimulated using the same stimulation protocol in cytokine release experiments. For endothelial cells, hCAECs were seeded on 6 well plates at density of 4 × 10^4^ cells per well, cells were allowed to attach overnight followed by DEX and MCC treatment (50 µm). At 24 and 48 h after drug treatment, RNA was collected. Gene expression analysis was modified with optimized annealing temperature for primers used in qPCR. Primers for pro‐ and anti‐inflammatory genes included MCP‐1, IL‐1β, CD206, and Fizz1. Primers for endothelial genes included CDH5, PECAM, eNOS and KDR. Please see Table S1 (Supporting Information) for full primer sequences.

Total RNA was extracted with TRI Reagent (Sigma) according to the manufacturers protocol. cDNA was reverse transcribed from RNA (1 µg) using SensiFast cDNA synthesis kit (BiolineQuantitative real time PCR was performed with Roche Light cycler LC480. PCR conditions were as follows: 95 °C for 2 min, 95 °C for 5 s, 58 °C for 30 s, and 72 °C for 20 s. PCR reactions were repeated for 45 cycles. 18S ribosomal RNA was used as a housekeeping gene for normalization. Expression levels were calculated by the relative quantification method (Delta‐Delta threshold cycle). Reverse‐transcription quantitative real‐time PCR was performed in triplicate for each sample.

### Myofibroblast Differentiation

hFBs were treated with transforming growth factor β (TGF‐β) to induce myofibroblast transdifferentiation.^[^ ^17^ ^]^ First, hFBs were seeded into 96‐well plates (3000 cells per well) and allowed to attach overnight. TGF‐β (2 ng mL^−1^) was added to each well and cells were incubated for 24 h. hFBs were then treated with DEX or MCC (50 µm) and incubated for 24 h (TGF‐β remained in cell media). Finally, hFBs were fixed and immunostained for SMα actin (1:50, Abcam, ab7817) and collagen type I (1:50, Abcam, ab2413) to measure the levels of myofibroblast differentiation.

### Western Blot

Collagen type 1 expression was further assessed using western blot. Using the same myofibroblast differentiation protocol above in a 12 well plate, protein lysates were extracted with Mammalian Cell Lysis Buffer (Sigma). Protein concentration was determined by a standard bicinchoninic acid assay (Thermo Fisher Scientific). Protein (5 µg) was loaded onto NuPAGE Novex 4–12% Bis‐Tris gels (Life Technologies) and separated using SDS‐PAGE electrophoresis. Protein was transferred to polyvinylidene fluoride membranes using a semidry iBlot gel transfer system (Life Technologies). Membrane was blocked with BSA (5%) in PBS for 1 h at room temperature. Primary antibody was incubated at 4 °C overnight in BSA (1%) at appropriate dilutions for Col I (1:500, Abcam, ab2413) and α‐tubulin (1:2000, Abcam, ab7291) as loading control. Membrane was washed with PBST (Tween20, 0.1%) followed by incubation with secondary antibody conjugated with horseradish peroxidase (HRP) for 2 h at room temperature in skim milk (1%) in PBS. Protein was detected using Luminata Crescendo Western HRP substrate (Millipore). Western blotting densitometry was analyzed using Image Lab Software 6.1 (Bio‐Rad).

### Endothelial Integrity and Function

The effects of DEX and MCC on endothelial integrity and function were assessed using hCAECs. Cells were seeded into 96‐well plates (3000 cells per well), allowed to attach overnight then treated with DEX or MCC (50 µm), and incubated for 3 days. Cells were then fixed (10% formalin), and immunostained for endothelial nitric oxide synthase (eNOS) (1:200, Abcam, ab33168) and vascular‐endothelial cadherin (VECad) (1:100, Abcam, ab76198),^[^ ^32^ ^]^ then imaged using a Zeiss Zen Z1 fluorescent microscope.

### Electrospinning Polycaprolactone Scaffolds

PCL scaffolds were manufactured by electrospinning (IME Medical Electrospinning, Waalre, Netherlands). PCL solution was prepared by dissolving PCL in 1,1,1,3,3,3‐hexafluoroisopropanol (HFP) at 10% w/v with overnight mixing at room temperature. Solution was loaded into a syringe (Terumo Corp., Tokyo, Japan) and pumped at 4 mL h^−1^ to a needle (20 G) with an applied voltage of 20 kV, separated by an air gap distance of 16 cm from a stainless steel drum (10 cm diameter) rotating at 500 rpm. Electrospun scaffolds were removed from the drum, washed and air dried to remove residual solvent, and cut into circular discs with a 6 mm diameter biopsy punch.

### Murine Subcutaneous Implant

Study was approved by the University of Sydney Animal Ethics Committee (protocol number 2020/1785). Experiments were conducted in accordance with the Australian Code of Practice for the Care and Use of Animals for Scientific Purpose. C57BL/6 mice (male, 9–10 weeks old, 25 ± 2 g), purchased from Animal BioResources (Moss Vale, NSW, Australia), were used for this model. Both drugs were evaluated in vivo using a subcutaneous mouse implantation model as previously described.^[^ ^18^ ^]^ Briefly, each mouse was implanted with three scaffolds spaced 2 cm apart subcutaneously across their back, a control (untreated) PCL scaffold, and separate scaffolds passively absorbed overnight at 4 °C with DEX or MCC950 (2.5 mm in PBS). At endpoints of day 3, 14, and 28 post‐implant, mice were euthanized and samples collected for histological processing.

### Bioluminescent Cell Tracking

Immune cell homing and engraftment to implanted scaffolds was non‐invasively tracked using bioluminescence imaging in an in vivo imaging system (IVIS; Perkin Elmer, USA) as previously described.^[^ ^18^ ^]^ Briefly, bone marrow‐mononuclear cells (BM‐MNCs) were harvested from the hindlimbs of donor FVB‐L2G transgenic mice (Jackson Laboratories, USA) expressing firefly luciferase and isolated using Ficoll density gradient centrifugation. BM‐MNCs were then injected (3 × 10^6^ cells in 200 µL) into the tail vein of C57BL/6 mice immediately after being implanted with scaffolds. Mice were serially imaged until day 14 post‐implantation. Bioluminescence was measured in units of radiance (photon s^−1^ cm^−2^ steradian^−1^) and quantified as mean radiance values within a predefined region of interest surrounding each scaffold. At endpoints of day 14 post‐implantation, mice were euthanized and samples collected for histological processing.

### Histology and Immunostaining

The explanted scaffolds were fixed overnight at room temperature with paraformaldehyde (4%). The samples were dehydrated using an ascending ethanol gradient, embedded in paraffin and sectioned at 5 µm. For histological staining, sections were deparaffinized, rehydrated, and stained using hematoxylin and eosin and Masson's trichrome. For immunohistochemistry staining, using primary antibodies against CD68 (1:500, Abcam, ab125212) for macrophage, neutrophil elastase (1:200, Abcam, ab68672) for neutrophils, vimentin (1:200, Abcam, ab45939) for fibroblast, CD31 (1:100, Abcam, ab182981) for endothelial cells, and smooth muscle α‐actin (1:500, Sigma, F3777) for smooth muscle cells. The sections were counter stain for nuclei using mounting media containing DAPI (Sigma, F6057) and imaged on Zeiss AxioScan microscope at 20× magnification.

### Image Analysis

Image analysis was done using ImageJ. For pyroptosis analysis, total cell number per image was quantified with particle counts for DAPI staining and cells with positive actin staining were counted manually. For in vitro experiments with immunohistochemistry, positive staining was quantified as a percentage of total area based on a common threshold.

For in vivo histological analysis, immunohistological analysis for neutrophils, macrophages, fibroblast and M1/M2 markers were quantified as a percentage of total scaffold area based on a common threshold. Fibrotic capsule thickness was measured manually at 3 different points on the scaffold in H&E staining. Capillary (CD31^+^ only) and arteriole (CD31^+^ and SMα^+^) density were counted manually for vessels with defined lumens in the area of interest at the site of the scaffold. For native arteriole formation, diameter of arterioles was measured manually across the largest measured diameter of an arteriole and counting those which were ≥20 µm (*n* = 4/5 sections per scaffold group per time point).

### Statistical Analysis

Analyses were performed in GraphPad Prism 9 (Graphpad Software, San Diego, California). Data are expressed as mean ± standard error of the mean (SEM). Statistical significance was determined by Student's *t*‐test for two group data sets, or one‐way analysis of variance (ANOVA) with post hoc Dunnett's multiple comparisons test for more than 2 group data sets or two‐way ANOVA with Tukey's multiple comparison test for more than 2 group data sets with multiple timepoints. *P* < 0.05 was considered statistically significant. *, **, ***, and **** display *p* < 0.05, *p* < 0.01, *p* < 0.001, and *p* < 0.0001 respectively.

## Conflict of Interest

The authors declare no conflict of interest.

## Supporting information

Supporting Information

## Data Availability

The data that support the findings of this study are available from the corresponding author upon reasonable request.
