# Supplementary material for: Selective Immunosuppression Targeting the NLRP3 Inflammasome Mitigates the Foreign Body Response to Implanted Biomaterials While Preserving Angiogenesis
Source: Adv Healthc Mater. 2023 Nov 1;12(32):2301571. doi: 10.1002/adhm.202301571 (PMC11469290; doi:10.1002/adhm.202301571)
Supplement: Supplementary file 1 — Supporting Information [file ADHM-12-2301571-s001.pdf]

ADVANCED HEALTHCARE MATERIALS

Supporting Information

for *Adv. Healthcare Mater.*, DOI 10.1002/adhm.202301571

Selective Immunosuppression Targeting the NLRP3 Inflammasome Mitigates the Foreign Body Response to Implanted Biomaterials While Preserving Angiogenesis

*Alex H.P. Chan, Matthew J. Moore, Angus J. Grant, Yuen Ting Monica Lam, Matthew V. Darnell, Praveesuda L. Michael, Steven G. Wise and Richard P. Tan**

Supplemental Figures

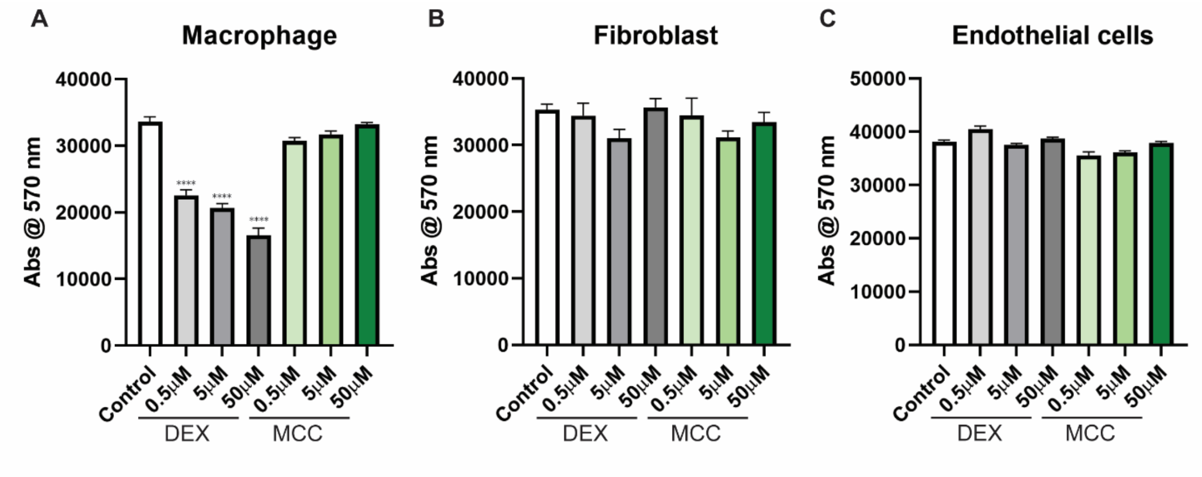

Figure S1: Cytotoxicity of DEX and MCC on macrophage, fibroblast and endothelial cells. alamarBlue cytotoxicity assay following 3 day culture with DEX and MCC950. A) Macrophages. B) Fibroblasts. C) Endothelial cells. Data represented as mean ± SEM (n = 3-4). Statistical significance was determined using Dunnett's multiple comparison one-way ANOVA test relative to control group (****$p < 0.0001$).

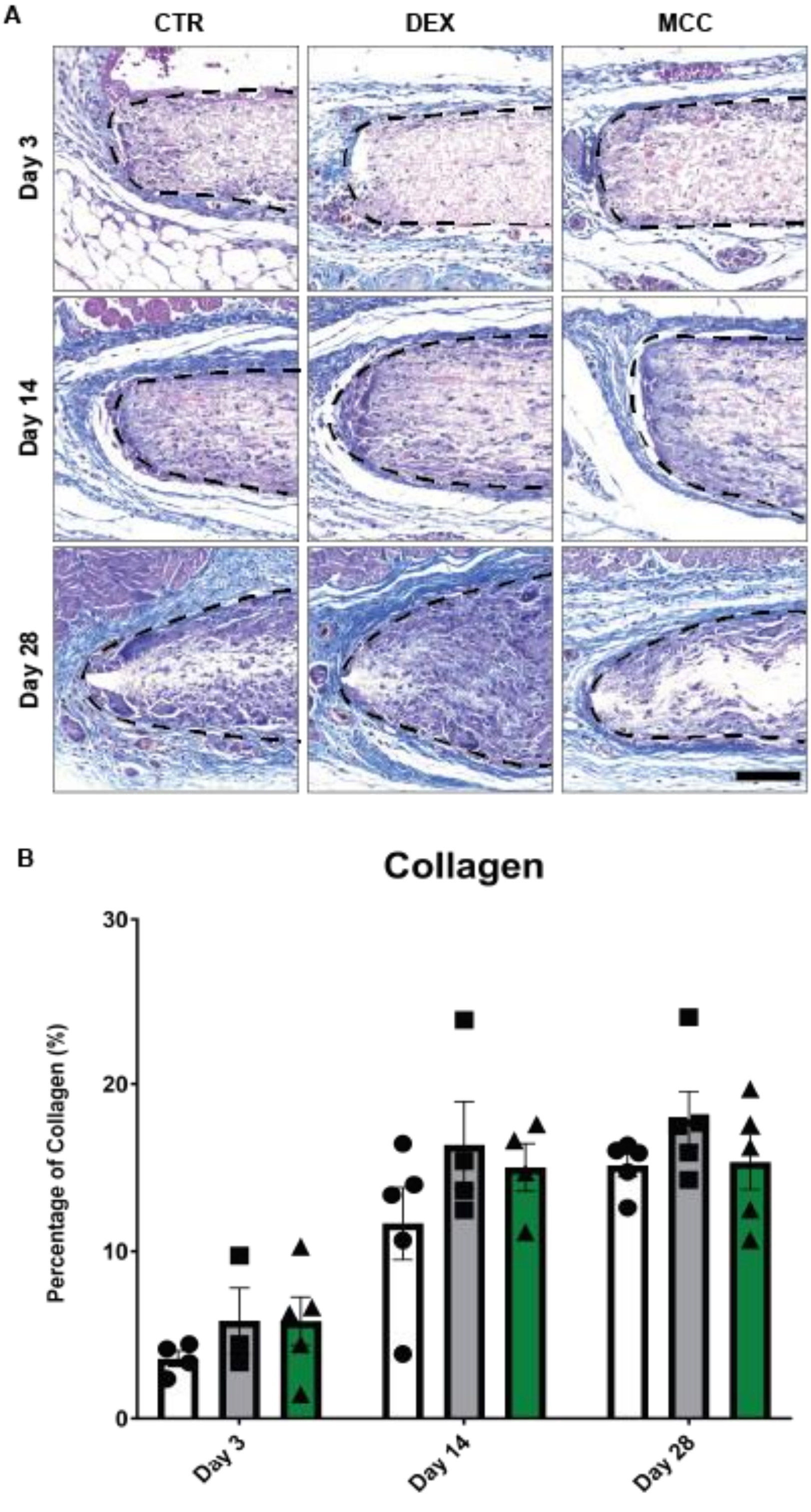

Figure S2: DEX and MCC950 did not significantly affect collagen content at the fibrotic capsule. A) Representative images of Masson's Trichrome staining, collagen shown in blue. B) Quantification of collagen represented as area percentage of collagen staining in the fibrotic capsule. Data represented as mean ± SEM (n = 4-5). Scale bar represents 100 µm.

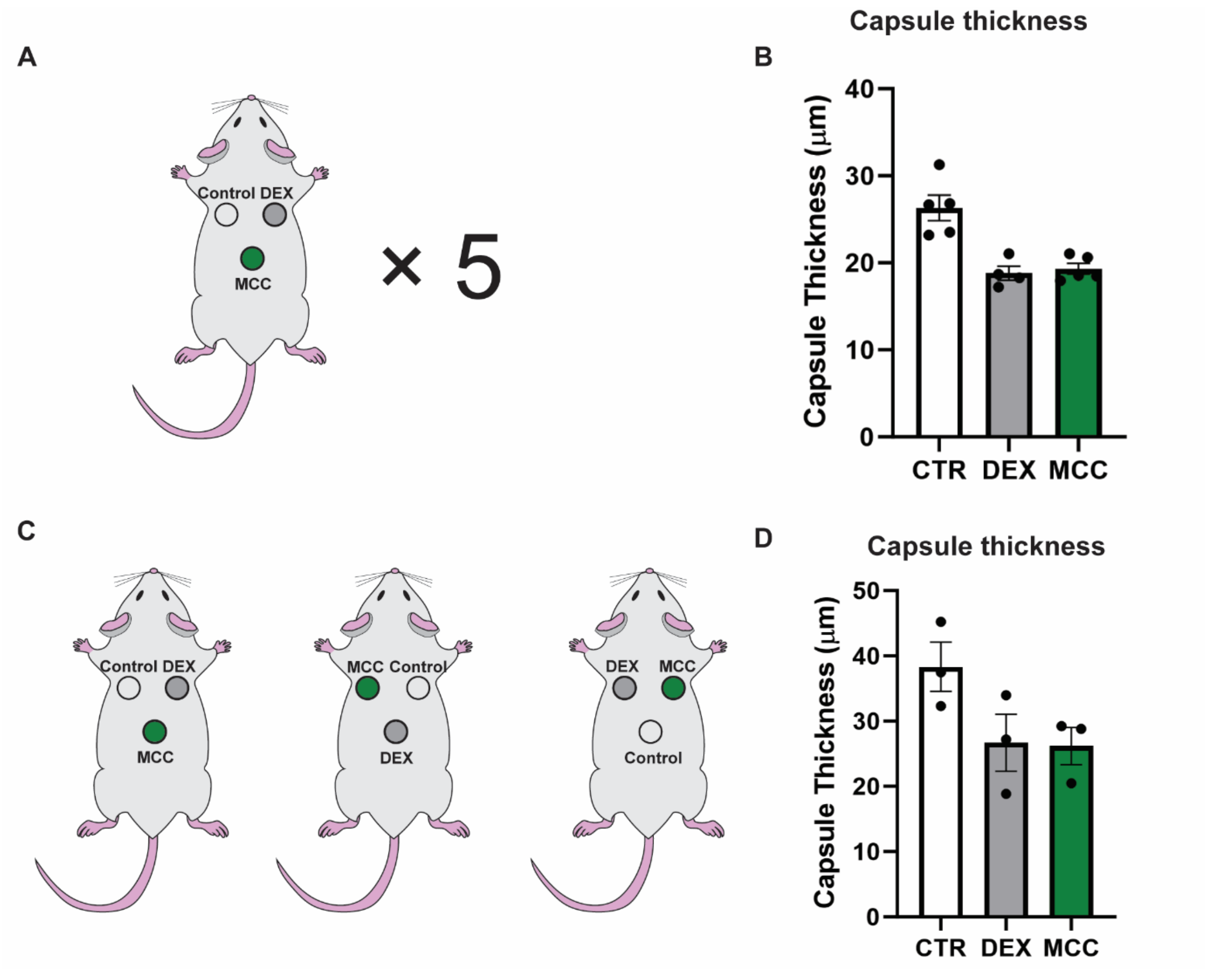

Figure S3: Scaffold proximity does not impact localised drug cross-reactivity to adjacent scaffolds. A) Non-rotated scaffold implantation positions, all mice received scaffold implantation in the same orientation. B) Quantification of fibrotic capsule thickness at day 14 with non-rotated scaffold positions. C) Rotated scaffold positions, a separated mice cohort with scaffolds implanted in different locations. D) Quantification of fibrotic capsule thickness at day 14 in separate cohort of mice each with rotated scaffold positions allowing for the implantation of each scaffold group in each subcutaneous location.

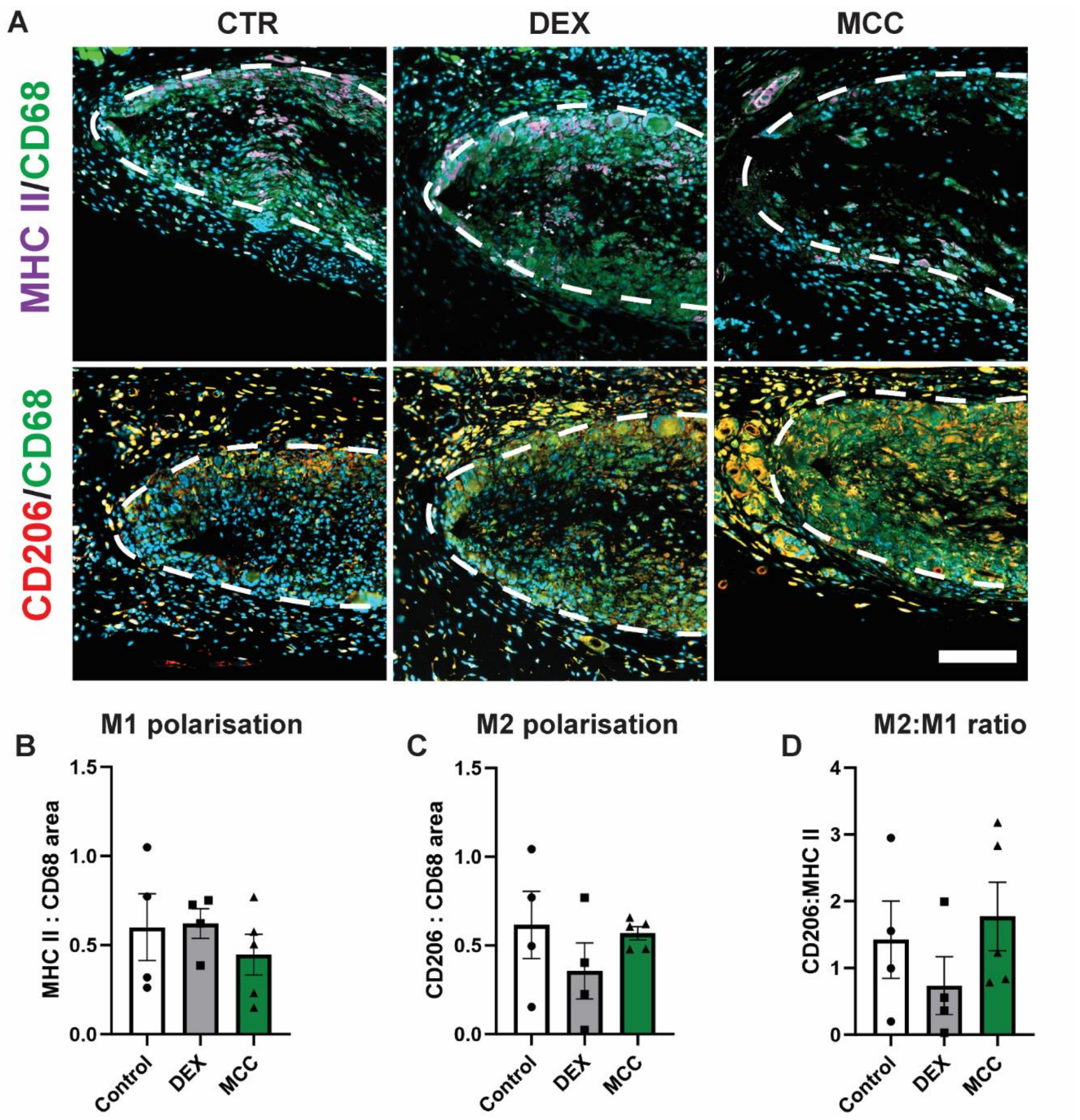

Figure S4: DEX and MCC950 did not significantly affect macrophage polarization. A) Representative images of macrophages stained with CD68 (green) and M1 marker, MHC II (purple) or M2 marker, CD206 (red), counterstained for nuclei with DAPI (blue). B) Quantification of M1 polarisation as represented by ratio of percentage area of MHC II and CD68. C) Quantification of M2 polarisation as represented by ratio of percentage area of CD206 and CD68. D) Quantification of M2/M1 macrophage polarisation as represented by ratio of CD206 and MHC II. Data represented as mean ± SEM (n = 4-5). Scale bar represents 100 μm.

Table S1: Primer sequences for qPCR.

Gene	Forward	Reverse
MCP1	GCTCAGCCAGATGCAGTTAA	TCTTGAGCTTGGTGACAAAAACT
IL1β	TGTAATGAAAGACGGCACACC	TCTTCTTTGGGTATTGCTTGG
CD206	CAGGTGTGGGCTCAGGTAGT	TGTGGTGAGCTGAAAGGTGA
Fizz1	CACCTCTTCACTCGAGGGACAGTTG	GGTCCCAGTGCATATGGATGAGACC
18S	GTAACCCGTTGAACCCCATT	CCATCCAATCGGTAGTAGGG
CDH5	CGCAATAGACAAGGACATAAC	TATCGTGTGATTATCCGTGAGG
PECAM1	AGATACTCTAGAACGGAAGG	CAGAGGTCTTGAAATACAGG
eNOS	CGGAGAATGGAGAGAGCTTTG	TGCTGTTGAAGCGGATCTTA
KDR	GTACATAGTTGTCGTTGTAGG	TCAATCCCACATTTAGTTC
